# Anti-malarial drug: the emerging role of artemisinin and its derivatives in liver disease treatment

**DOI:** 10.1186/s13020-021-00489-0

**Published:** 2021-08-18

**Authors:** Ye Xiong, Jianrong Huang

**Affiliations:** The Department of Infectious Diseases, State Key Laboratory for Diagnosis and Treatment of Infectious Diseases, National Clinical Research Center for Infectious Diseases, Collaborative Innovation Center for Diagnosis and Treatment of Infectious Diseases, The First Affiliated Hospital, Zhejiang University School of Medicine, 79 Qingchun Road, Hangzhou, 310003 China

**Keywords:** Artemisinin, Liver disease, Malaria, Anti-inflammatory, Apoptosis

## Abstract

Artemisinin and its derivatives belong to a family of drugs approved for the treatment of malaria with known clinical safety and efficacy. In addition to its anti-malarial effect, artemisinin displays anti-viral, anti-inflammatory, and anti-cancer effects in vivo and in vitro. Recently, much attention has been paid to the therapeutic role of artemisinin in liver diseases. Several studies suggest that artemisinin and its derivatives can protect the liver through different mechanisms, such as those pertaining to inflammation, proliferation, invasion, metastasis, and induction of apoptosis and autophagy. In this review, we provide a comprehensive discussion of the underlying molecular mechanisms and signaling pathways of artemisinin and its derivatives in treating liver diseases. Further pharmacological research will aid in determining whether artemisinin and its derivatives may serve as promising medicines for the treatment of liver diseases in the future.

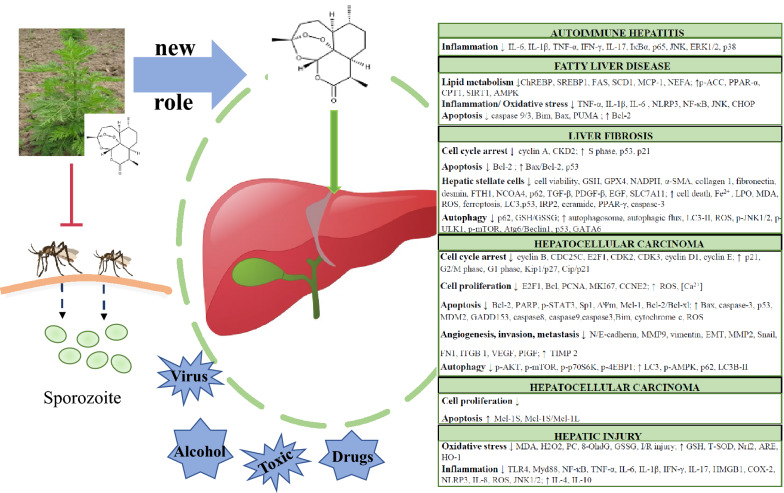

## Background

Liver diseases and their associated complications are major public health concerns and among of the most significant causes of death worldwide, owing to their progressive pathogenesis and lack of effective treatment modalities. Liver diseases include viral and non-viral hepatitis, alcoholic fatty liver disease (AFLD), non-alcoholic fatty liver disease (NAFLD), cholestatic liver diseases, autoimmune liver diseases, liver failure, liver fibrosis, and increasing end-stage liver disorders, such as cirrhosis and primary hepatic tumors, including hepatocellular carcinoma (HCC) and cholangiocarcinoma (CCA) [1–3]. As the main organ for detoxification and metabolism, the liver is important in protecting against toxicity. However, it is also vulnerable to various threats from the environment [4]. According to the World Health Organization’s (WHO) 2015 Global Health Estimates data, there were more than two million global deaths due to liver diseases in 2015, and hepatitis B virus-induced liver cirrhosis was the main cause of liver-related deaths in the Asia–Pacific region [5]. To date, NAFLD is rapidly becoming a predominant cause of liver-related morbidity and mortality worldwide [6]. However, the clinical treatment of these chronic liver diseases is complicated by numerous problems, including progressive disease courses, late detection, and limited treatment options. Once chronic liver disease progresses to decompensated liver cirrhosis and end-stage HCC, treatments become increasingly limited. Therefore, it is of great importance to explore alternative effective drugs.

Traditional Chinese herbal medicine (TCHM) has been widely used to treat various diseases for thousands of years in China and throughout the world, and the efficacies of many treatments have been verified by modern science [7]. For example, herbal compounds such as curcumin, berberine, artemisinin, and Honeysuckle-derived microRNA have been verified to display anti-cancer activities through different molecular mechanisms [8, 9]. Additionally, a study showed that TCHM treatments played a positive role against the novel coronavirus disease (COVID-19), a worldwide pandemic caused by the virus SARS-CoV-2 [10]. The TCHM-based medication Cerebralcare Granule®, containing diverse active ingredients (such as Angelica sinensis, Chuanxiong Rhizoma, Radix Paeoniae Alba, etc.), performed well in the treatment of cognitive dysfunction, and Jinqi Jiangtang, a TCHM prescription composed of coptidis rhizoma, astragail radix, and lonicerae japonicae flos extracts, was beneficial for type 2 diabetes treatment [11, 12]. Increasing evidence shows that some TCHM prescriptions and single herb medicines play a prospective role in treating liver diseases. Silymarin, a flavonoid antioxidant derived from the plant *Silybum marianum* showed promise as a treatment for chronic liver diseases [13]. The Fufang Biejia Ruangan pill, the first clinically approved herbal anti-fibrosis formula in China, has been shown to prevent tumors in mice [14]. However, it was reported that ingesting traditional and herbal medicines was a major cause of drug-induced liver injury in South Korea over a 2-year period across 17 hospitals [15]. Further research should be conducted to discover effective treatments without harmful effects.

Artemisinin, or qinghaosu, is a sesquiterpene trioxane lactone with a peroxide bridge [16]. It is derived from *Artemisia annua L.*, an herb which has been used for the treatment of fevers and chills for more than a thousand years [17]. In the 1950s, the increasing resistance of *Plasmodium falciparum* to existing antimalarial drugs led scientists in various countries to research for better therapeutic approaches against malaria. Chinese researchers turned to an investigation of traditional medicines, and found that *Artemisia annua L.* exhibited a strong inhibitory effect against *P. falciparum*[18]. Inspired by the ancient book Ge Hong zhou hou bei ji fang (Prescriptions for Emergencies), they extracted the plant’s active component and were able to provide artemisinin to countries devastated by malaria [19]. Youyou Tu from the China Academy of Traditional Chinese Medicine even one the 2015 Nobel Prize in Medicine for the discovery [18].

However, with continued research, artemisinin was found to be poorly soluble in both oil and water and to exhibit a short plasma half-life, limiting its therapeutic value [20]. Dihydroartemisinin (DHA) was developed a first-generation derivative by modifying the carbonyl groups into hydroxyl groups [21]. It is a more effective and stable antimalarial drug than artemisinin. Similarly, converting DHA into methyl and ethyl ether produced the more oil-soluble artemether (ARM) and arteether (ARE), respectively [22]. The smaller molecule artesunate (ART) was developed as a more water-soluble hemisuccinate derivative. Compared to artemisinin, these derivatives are more effective and easier to produce [21]. Recently, new derivatives (SM1044, SM905, SM934, and AM735) [23] have been developed, bringing interesting possibilities for future research. Detailed information regarding artemisinin and its derivatives is shown in Table 1 [24–32].

**Table 1:** Characteristics of artemisinin and its derivatives

Derivatives	Molecular formulas	Chemical structures	Structural modifications	Physical properties	Formulations	Route of administration	Pharmacokinetics	Pharmacological activities	Ref
Artemisinin	C_15_H_22_O_5_		-	Poorly soluble in water and oil; Melting point 156–157℃; Molecular weight 282.35	Powder; Micronized powder; Tablet; Capsule; Suppository	Oral Intramuscular injection Rectal	T_max_ 1–3 h Volume of distribution 1420–1560L Clearance 445–479L/h T_1/2_ 2.27–2.29 h T_max_ 3.4 h T_max_ 5.6 h	Antimalaria; Antivirus; Antihelminth; Anticancer; Antiinflammation;	[24–26, 31, 32]
DHA	C_15_H_24_O_5_		Modifying carbonyl groups into hydroxyl groups; The active metabolite of all artemisinins	Molecular weight 284.4	Powder; Tablet;	Oral Intramuscular injection Rectal	T_max_ 0.9–1.6 h – T_max_ 4 h	Antimalaria; Anticancer;	[24, 26, 28, 29, 32]
ART	C_19_H_28_O_8_		Hemisuccinate derivative of DHA	Water-soluble; Molecular weight 384.4	Powder Suppository Tablet Injection	Oral Intramuscular injection Rectal Intravenous administration	Bioavailability 61%-88% T_max_ 15–39 min Volume of distribution 14.8L/kg Clearance 20.6L/kg/hr T_1/2_ 0.36–1.2 h Bioavailability 86.4–88% T_max_ 7.2–12 min T_1/2_ 25.2–48.2 h Clearance 2.4–3.48L/kg/hr Volume of distribution 1.09–3.98L/kg Bioavailability 54.9% T_max_ 0.58–1.43 h T_1/2_ 0.9–0.95 h Clearance 2–3L/kg/hr Volume of distribution 0.1–0.3L/kg	Antimalaria; Antiparasite; Antitumor; Antivirus; Antiinflammation; Antibacterial	[24, 27, 30–32]
ARM	C_16_H_26_O_5_		Production methyl ether from DHA	Oil-soluble; Molecular weight 298.4	Powder Injection Capsule	Oral Intramuscular injection Rectal	T_max_ 1.7–6 h T_max_ 1.3–8.7 h T_max_ 3.1 h	Antimalaria	[24, 31, 32]
ARE	C_17_H_28_O_5_		Production β-ethyl ether from DHA	Oil-soluble; Practically insoluble in water; Molecular weight 312.4	Powder Injection	Oral Intramuscular injection Rectal	– T_max_ 4.8–7 h –	Antimalaria	[24, 31, 32]

*T*_*max*_ Time to maximal concentration, *T*_*1/2*_ Elimination half-life

Through extensive research on artemisinin, researchers revealed that its primary anti-malarial activity is due to special internal structures called endoperoxide linkages (peroxide bridges) [33]. When the malaria parasites invade the human body, they digest a large amount of hemoglobin in host erythrocytes to obtain the nutrients necessary for growth and maturation. Hemoglobin digestion releases abundant heme and free ferrous iron. This heme and iron activate artemisinin, cleaving the peroxide bridge and producing free radicals that alkylate malaria membrane-associated proteins and impair mitochondria functions, as well as reactive oxygen species (ROS), which induce parasite damage and eventual death [20, 34–37]. A study also found that deoxyartemisinin, a substance lacking peroxide bridges, cannot induce the above process [22, 38], verifying that the endoperoxide linkages in artemisinin are vital to its efficacy.

In recent years, numerous studies showed that artemisinin and its derivatives also play a significant role in treating other diseases, apart from malaria. Artemisinin regulates inflammation and apoptosis, which, together with antioxidant effects, supports an argument for the use of artemisinin in different pathologies. ART inhibits human cytomegalovirus infection by antagonizing nuclear factor kappaB (NF-κB) and downstream activities of AKT1 and p70S6K [39]. DHA may be used to treat systematic lupus erythematosus-related nephritis by blocking the NF-κB signaling pathway, increasing tumor necrosis factor-α (TNF-α) secretion, and reducing the production of anti-ds-DNA antibodies [37]. Research has also reported that ART regulates glucose levels [40, 41].

Recently, a growing number of TCHM treatments and their active ingredients have been discovered to have hepatoprotective properties [42–45]. Most of them, however, are toxic to some extent, and the underlying molecular mechanisms and refining technologies remain poorly understood. Artemisinin is thought to have be one of these hepatoprotective treatments has been widely used in ancient times to reduce fever and eliminate jaundice and hepatitis [46]. Because artemisinin has consistently been considered safe and effective in clinical practice and was officially recommended by WHO in the 1980s, it is likely among the less toxic TCHM treatments with hepatoprotective effects [47]. This applies to artemisinin derivatives as well, with one study showing that an oral dose of 2.2–3.9 mg/kg/d ART (add-on oncological therapy) is safe and well tolerated by subjects with metastatic breast cancer, without altering kidney function, liver function, or routine blood tests [48]. As opposed to other TCHM treatments with unclear hepatoprotective mechanisms, artemisinin and its derivatives are known to protect the liver through antioxidant, anti-inflammatory, pro-apoptotic, and carcinostatic mechanisms. The structure of endoperoxide linkage and production of ROS is not only regarded as a vital feature for antimalarial treatment, but is also key to liver protection [49]. Therefore, artemisinin deserves further examination to determine its clinical usage. Although artemisinin may play a promising role in liver disease treatment, no review has been conducted to systematically clarify the potential use and mechanism of artemisinin and its derivatives in the treatment of liver diseases.

This review serves to discuss the hepatoprotective effects of artemisinin and its derivatives, ART, DHA, ARM, and ARE, in vitro and in vivo.

## Pharmacological effects of artemisinin and its derivatives in vitro

### Inducing cell cycle arrest

Emerging evidence has indicated that artemisinin and its derivatives induce cell cycle arrest at different phases in HCC and non-HCC cells. Zhang and colleagues demonstrated that all three types of HCC cells, HepG2, PLC/PRF/5, and Hep3B cells, were apparently arrested in the G2/M phase following DHA treatment, with reduction in the levels of cyclin B and cell division cycle 25 homolog C (CDC25C), and induction of p21 [50]. Similarly, ART and DHA induced both p53 wild-type (HepG2) and p53 null (Hep3B) cell G1-phase cell cycle arrest. This process was mediated by increased Cip1/p21 and Kip1/p27 protein expression, with significant decreases in E2F transcription factor 1 (E2F1), cyclin-dependent kinase (CDK) 2, CDK3, cyclin D1, and cyclin E [51]. On the contrary, DHA dose-dependently downregulated the expression of cyclin A and CDK2, and increased p53 and p21 expression in hepatic stellate cells (HSCs). Treatment with DHA for 24 h induced S-phase accumulation in HSCs [52]. A relatively lower dose of ART significantly inhibited the proliferation of normal rat liver BRL-3A and mouse liver AML12 cells by inducing cell cycle arrest at the G0/G1 phase, with corresponding downregulation of CDK4, cyclin D1, CDK2, and cyclin E1 expression [53]. Together, these studies suggest that artemisinins induce cell cycle arrest not only in HCC cells, but in normal, healthy liver cells. Thus, although previous research indicates that artemisinins can restrain the growth of tumor cells by blocking the cell cycle, more attention should be paid to the possible adverse effects of artemisinin-based drugs on healthy normal cells when developing therapies.

### Inhibition of hepatic oxidative stress and inflammation

Some studies reported that artemisinins exert anti-inflammatory effects in vitro. During plating of HSCs exposed to platelet derived growth factor (PDGF)-BB in vitro, we observed that PDGF-BB induced the production of NLR family pyrin domain containing 3 (NLRP3), pro-interleukin (IL)-1β, pro-IL-4, pro-IL-18, interferon (IFN)-γ, and IL-1β. Treatment with DHA inhibited the expression of pro-inflammatory factors in a dose-dependent manner [54].

### Inhibition of cell proliferation

The anti-proliferation effects of artemisinin and its derivatives are mostly observed in HCC cell lines. DHA inhibited the proliferation of human HCC cells (HepG2215 cells) in a dose- and time-dependent manner [55], and 24-h treatment yielded obvious inhibitory effects [56]. DHA also played a significant role in inhibiting the proliferation of HUCCT-1 and FRH201 cells. The inhibitory effect began after 12 h, suggesting a time-dependent mechanism of action [57]. Similarly, DHA reportedly inhibited the proliferation of HepG2 cell lines by inducing the intracellular production of ROS and [Ca^2+^] [58]. The proliferation of HCCLM6 cells was effectively inhibited after DHA treatment for 48 h [59]. By evaluating the anti-proliferative activity of ART, we concluded that it significantly reduced the clonogenic ability of HCCLM3 and MHCC97H cells in a dose-dependent manner [60]. Moreover, artemisinin and its derivatives displayed anti-proliferative activity in HepG2 and SK-HEP-1 cells. However, the anti-proliferative effects differed across different derivatives. Interestingly, 10-dihydroartemisinyl 2’-propylpentanoate exerted stronger anti-proliferative effects on the two cell lines assayed, and this effect was five-fold more potent than that of sorafenib [61, 62].

Further, the anti-proliferative effects of artemisinin and its derivatives not only occur in cancer cells, but also in non-cancer cells. ART strongly inhibited proliferation of activated rat primary HSCs and LX-2 cells in a dose- and time-dependent manner in vitro [63, 64].

### Apoptosis induction

The apoptotic effects of artemisinin and its derivatives are clearly observed in HCC cell lines. ART affected the progression of apoptosis in LX-2 cells, and ART treatment reduced B-cell lymphoma (Bcl)2 expression and increased the bcl-2-associated X (Bax)/Bcl-2 ratio in a dose-dependent manner [64]. Another study demonstrated that ART increased p53 at both the mRNA and protein levels, inducing apoptosis in rat primary HSCs [63]. Artemisinin induced apoptosis in SMMC-7721 cells in a time- and dose-dependent manner [65]. ART and DHA induced apoptosis in HepG2 and Hep3B cells. Lv et al.showed that treating HepG2 and Hep3B with ART and DHA induced an increase in the expression of Bax protein and a decrease in the expression of Bcl-2 [51]. The increased Bax/Bcl-2 ratio promoted cytochrome c to enter the cytosol and combine with apoptotic protease activating factor-1 (Apaf-1), activating caspase-3 and poly ADP-ribose polymerase (PARP). Furthermore, the study also demonstrated that treatment of HepG2 cells with ART and DHA resulted in decreased mouse double minute 2 (MDM2) and increased p53. Generally, p53 is one of the mediators of the mitochondrial apoptotic pathway [66–68]. Interestingly, in p53-null Hep3B cells, we observed the same apoptotic phenomenon, indicating that the ART- and DHA-induced mitochondrial apoptotic pathway can be both p53 dependent and independent [51]. The signal transducers and activators of transcription (STAT)3-signaling pathway are upstream of apoptosis inhibitor genes; thus, inhibition of STAT3-signaling pathway and increased levels of factor related apoptosis (Fas) on the surface of HepG2 cells promotes apoptosis. Therefore, ART treatment induces apoptosis of HepG2 cells [69]. Another research team also revealed that ART could modulate STAT3-dependent anti-apoptotic and pro-apoptotic expression. Treatment with ART evidently promoted apoptosis of HCC cells, activation of procaspase-3, suppression of Bcl-xL, and survivin [70]. DHA similarly induced apoptosis in HepG2, PLC/PRF/5, and Hep3B cells. Zhang and colleagues observed that caspase 9 and 3, but not caspase 8, largely induced apoptosis in cells exposed to DHA. Additionally, mitochondrial membrane depolarization, PARP cleavage, and cytochrome c release all implied that DHA may induce apoptosis in HCC cells via the intrinsic mitochondrial pathway. However, increased apoptosis was observed in wild-type p53-expressing cells compared to two other cell types, which suggested that p53 may play a role in promoting apoptosis [50]. DHA was found to induce ROS-mediated apoptosis in HepG2 cells in a dose- and time-dependent manner, together with increased intracellular [Ca^2+^] concentrations. Treatment of HepG2 cells with DHA increased Bax protein, growth-arrest-and-DNA-damage-inducible gene 153 (GADD153), and apoptotic pathway-related protein levels, but decreased Bcl-2 protein levels [58]. A hybrid of ursodeoxycholic acid and DHA also induced ROS-mediated apoptosis in HepG2 cells, resulting in the upregulation of caspase-3 and cleaved PARP. This hybrid also induced apoptosis at a much lower concentration in HCC cells than DHA alone [71]. Moreover, DHA induced caspase-dependent apoptosis in SK-Hep-1 cells by inhibiting the Sp1 pathway and activating caspase 8, 9, and 3 [72]. DHA also induced apoptosis in HepG2 and Huh-7 cells via a ROS-dependent and Bcl-2 interaction mediator of cell death (Bim)-mediated intrinsic pathway with downregulated myeloid cell leukemia-1 (Mcl-1), while releasing bcl-2 homologous antagonist/killer (Bak) but not Bax [73]. Similarly, Pang and colleagues observed Bax activation, mitochondrial outer membrane permeabilization, cytochrome c release, and caspase 9 and 3 activation. ART also induced apoptosis in a ROS- and Bax-mediated pathway in Hep3B and Huh-7 cells [74]. However, contrary observations were recorded by Qin and colleagues, who reported that ROS induced by ART could not mediate apoptosis in HepG2 cells. Furthermore, ART induced apoptosis via the Bax-mediated intrinsic pathway in which Bcl-2/-xl was involved, but Bim, p53 upregulated modulator of apoptosis (Puma), and Mcl-1 were not involved [75]. In a recent study, ART treatment not only induced the expression of the intrinsic apoptotic markers Bax and Bcl-2, but also increased the levels of caspase-3/7 and cleaved PARP1 in HCC cells (HuH-7 cells and PLC/PRF/5 cells). A combination of sorafenib, a targeted drug for the treatment of liver cancer, with ART exhibited a more significant pro-apoptotic effect [76]. Further studies showed that QBC939, HUCCT-1, and FRH0201 cells exhibited increased apoptotic activity following DHA treatment, which was accompanied by the increased expression of Mcl-1S protein and the increased ratio of Mcl-1S/Mcl-1L [57, 77]. Taken together, artemisinin and its derivatives can induce apoptosis in various cells and through very different pathways within the same cell lines.

### Inhibition of angiogenesis, invasion, and metastasis

Tumor angiogenesis, invasion, and metastasis are principal methods through which cancer cells spread, resulting in high malignance and high mortality of HCC. Epithelial-to-mesenchymal transition (EMT) is a process whereby epithelial cells lose cell–cell adhesion and cell polarity and gain migratory, invasive, and anti-apoptotic abilities to obtain a mesenchymal phenotype [78]. Therefore, it has been regarded as the initiation of metastasis during cancer progression in many recent studies [79, 80]. In SK‐HEP1, SM7721, HepG2, and Huh7 HCC cell lines, ART significantly suppressed the occurrence of EMT by decreasing N/E-cadherin, matrix metalloproteinase (MMP)9, and vimentin. According to the analysis of clinical samples in vitro, ART could inhibit EMT, migration, and metastasis by decreasing the expression of LncRNA RP11. Besides, ART directly inhibited invasion and migration of SK-Hep1 and SM7721 cells [81]. Using wound healing and transwell assays, researchers observed that DHA significantly reduced the migration of HepG2215 cells [55], while ART inhibited the invasion and migration of HCCLM3 and MHCC97H cells. Furthermore, HCC cells exposed to ART showed downregulation of pro-metastatic and pro-invasive protein N-cadherin, MMP2, MMP9, and Snail, while the expression of anti-metastatic protein E-cadherin was upregulated, suggesting that ART may suppress the migration and invasion of HCC cells by adjusting the N-cadherin-snail-E-cadherin axis [60]. DHA also inhibited the migration and metastasis of HCCLM6 cells by downregulating fibronectin-1 (FN1) and β1-integrin (ITGB1) via the phosphoinositide 3-kinase (PI3K)/AKT signal pathway in vivo and in vitro [59]. Other researchers observed that ART could effectively suppress invasion and metastasis of HepG2 and SMMC7721 cells in vitro, coupled with downregulation of MMP2, upregulation of tissue inhibitor of metalloproteinases (TIMP)2, and degradation of the extracellular matrix (ECM). ART enhanced the adhesion of HCC cells through cell division cycle 42 (Cdc42), thereby reducing metastasis [82]. Another study showed that ART reduced the angiogenic factors vascular endothelial growth factor (VEGF) and placental growth factor (PIGF) in vitro and in vivo, suggesting that ART may play a role in inhibiting angiogenesis [83]. Similarly, combination of ART and sorafenib suppressed migration of Huh7 and HepG2 cells [84]. In total, artemisinin and its derivatives have the potential to inhibit angiogenesis, invasion, and metastasis, mainly in relation to cancer cells. Therefore, whether similar effects are observed in non-cancer liver diseases remains to be explored.

### Suppression of HSCs

HSCs are mesenchymal cells that retain features of resident fibroblasts and pericytes [85]. In the presence of various liver injuries, quiescent HSCs are activated, suggesting the beginning of fibrosis, followed by cirrhosis and HCC [86]. ART was shown to inhibit the activation of primary mouse HSC cells induced by CCl_4_, and its effect was associated with ferroptosis and activation of ferritinophagy [87]. Wang and colleagues considered that this effect was associated with a p53-dependent mechanism. Meanwhile, the expression of tumor growth factor-β receptor 1 (TGF-βR1), PDGF-β receptor, and epidermal growth factor receptor (EGFR) 31–33 was inhibited after ARM treatment [88]. Similarly, ARM induced the death of HSCs via the ferroptosis pathway, through the action of STIP1 homology and U-box containing protein 1 (STUB1), an enzyme mediating the ubiquitin process of iron regulatory protein 2 (IRP2). ARM inhibited IRP2 binding, thereby increasing IRP2 accumulation in cells, leading to iron accumulation and ROS production [89]. Another study observed that ART weakened the expression of α-smooth muscle actin (α-SMA) and collagen I, which were generally regarded as markers of HSC activation, via inhibition of the FAK/AKT/β-catenin pathway [64]. ART ameliorated HSC activation through the ceramide synthesis pathway and promoted peroxisome proliferators-activated receptors-γ (PPAR-γ) and caspase-3 upregulation, as well as hydroxyproline downregulation [90].

In addition, ART influenced HSC activation by reducing the expression of mitochondrial complex I subunit NDUFB8 and complex III subunit UQCRC2. ART attenuated the mitochondrial function in HSCs, and this effect could not be seen in the liver-derived cells [91].

### Chemosensitization to chemotherapeutic agents

Drug resistance and tumor recurrence have long been major challenges for cancer treatment [92]. Nevertheless, several studies held that artemisinin and its derivatives could promote sensitivity to other chemotherapeutic agents. Sorafenib (2.77 µM) combined with ART was equivalent to 5.23 µM sorafenib alone, and induced 50% inhibition of SK-7721 tumor cells. Moreover, sorafenib combined with ART treatment stimulated RAF/mitogen-activated protein kinase (MAPK) and PI3K/AKT/mammalian target of rapamycin (mTOR) signaling pathways, a dual inhibitory effect which promoted apoptosis [93]. Similarly, ART advanced cell apoptosis by activating the caspase cascade when combined with sorafenib, and using sorafenib and ART together reduced VEGFR2 protein expression in HepG2 and Huh7 cells [84]. DHA coupled with gemcitabine activated Bax-dependent apoptosis and decreased HepG2 and Hep3B cell survival [51]. Yang et al. showed that mutant p53 (R248Q) induced doxorubicin (ADM) resistance in Hep3B by increasing ADM efflux, AKT, extracellular signal-regulated protein kinases (ERK)1/2, and p65 phosphorylation and P-glycoprotein (P-gp) expression [94]. However, DHA enhanced the pro-apoptotic effects of ADM in Hep3B cells with mutant p53 (R248Q) synergistically, and it was indicated that DHA suppressed the P-gp expression by monitoring the p53 (R248Q)-ERK1/2-NF-κB pathway [95]. Taken together, these studies demonstrated that artemisinins can enhance therapeutic effectiveness of chemotherapeutic drugs through various mechanisms.

### Inducing autophagy

Autophagy is a process involving damaged cell removal, whereby cells eat themselves. It is a crucial for development, differentiation, and survival [96]. Autophagy is an important mechanism used to maintain the stability of intracellular environments, and can respond to various environmental and cellular stresses by mediating lysosome-dependent cell degradation processes [97]. Autophagy may occur in physiological and pathological conditions, indicating that regulated autophagy may serve as a promising therapeutic strategy [98]. Zhang and colleagues found that DHA induced the emergence of autophagosomes and increased autophagic flux in a dose- and time-dependent manner in activated HSCs, while downregulating p-mTOR activity and upregulating p-Unc-like kinase 1 (ULK1) activity [54, 99]. Similarly, in HepG2215 cells, DHA treatment markedly upregulated light chain 3 (LC3) and downregulated p-AKT, p-mTOR, p-ribosomal protein S6 kinase (p70S6K), and p-4E binding protein 1 (4EBP1), which suggested that DHA may induce autophagy via suppression of the AKT-mTOR pathway [55]. Additionally, LC3 beta (LC3B) conversion and autophagy substrate protein p62/sequestosome 1 (SQSTM1) are used to monitor autophagy [100]. DHA promoted the transformation from LC3B-I to LC3B-II and increased the expression of LC3B-II. Conversely, it induced the decreased p62/SQSTM1 in HepG2215 cells [56]. Some studies found that autophagy exerts opposing effects dependent on the cell type [101], which should be of particular concern in the future when using artemisinins to treat liver diseases. The pharmacological effects of artemisinins in vitro models are summarized in Table 2.

**Table 2:** Pharmacological effects of artemisinin and its derivatives on hepatic diseases in vitro

Hepatic disease	Cell lines	Drug	Dosage	Variations	Refs.
Effects on cell cycle					
HCC	HepG2 cells, PLC/PRF/5 cells, Hep3B cells	DHA	20–40 μM	↓cyclin B, CDC25C; ↑P21, G2/M phase	[50]
	HepG2 cells, Hep3B cells	ART, DHA	0–50 μM	↓E2F1, CDK2 , CDK3, cyclin D1, cyclin E; ↑G1 phase, Kip1/p27, Cip/p21	[51]
Liver fibrosis	HSCs	DHA	15–50 μM	↓cyclin A, CDK2; ↑S phase, p53, p21	[52]
Effects on cell proliferation					
HCC	HepG2215 cells	DHA	50–200 μM	↓cell proliferation	[55]
	HepG2215 cells	DHA	5–20 μM	↓cell proliferation, colony formation	[56]
	HepG2 cells	DHA	0–200 μM ol/L	↓cell proliferation; ↑ROS, [Ca^2+^]	[58]
	HCCLM6 cells	DHA	1–100 μM	↓cell proliferation, E2F1, BCL, PCNA, MKI67, CCNE2	[59]
	HCCLM3 cells, MHCC97H cells	ART	0–100 μM	↓cell growth, colony formation; cell cycle arrest	[60]
CCA	HUCCT-1 cells, FRH201 cells	DHA	20 μM ol/L	↓cell proliferation	[57]
Liver fibrosis	HSCs	ART	125–225 μM ol/L	↓cell proliferation	[63]
	LX-2 cells	ART	0–50 ug/ml	↓cell proliferation	[64]
Effects on cell apoptosis					
Liver fibrosis	LX-2 cells	ART	12.5–50 ug/ml	↓Bcl-2; ↑Bax/Bcl-2	[64]
	HSCs	ART	150–200 μM ol/L	↑p53	[63]
HCC	SMMC-7721 cells	Artemisinin	100–200 μM ol/L	↑apoptotic rate	[65]
	HepG2 cells	ART and DHA	0–50 μM	↓Bcl-2, PARP; ↑Bax, caspase-3, p53, MDM2	[51]
	HepG2 cells	ART	0.5–8 mg/L	↓p-STAT3; ↑Fas	[69]
	HepG2, cells	DHA	0–200 μM ol/L	↓Bcl-2; ↑GADD153, Bax,	[58]
	HepG2 cells	DHA	0–100 μM	↑cleaved PARP, caspase-3	[71]
	SK-Hep-1 cells	DHA	20–60 μM	↓PARP, Sp1; ↑caspase8, caspase9, caspase3	[72]
	HepG2 and Huh-7 cells	DHA	0–150 μM	↓ΔΨm, Mcl-1; ↑Bim, cytochrome c, caspase8, caspase9,caspase3, ROS, Bak	[73]
	Hep3B and Huh-7 cells	ART	10–150 μM	↑ROS, Bax, MOMP, cytochrome c, caspase 9, caspase3	[74]
	HepG2 cells	ART	10–150 μM	↓ΔΨm, Bcl-2/Bcl-xl; ↑Bax, caspase8, ROS, caspase9, caspase3	[75]
	HuH-7 cells and PLC/PRF/5 cells	ART	1–300 μM	↓Bcl-2; ↑Bax, caspase-3/7, cleaved PARP1	[76]
CCA	QBC939 cells, HUCCT-1 and FRH201 cells	DHA	20 μM ol/L	↑Mcl-1S, Mcl-1S/Mcl-1L, apoptotic rate	[57, 77]
Effects on angiogenesis, invasion, metastasis					
HCC	SK‐HEP1, SM7721, HepG2, and Huh7 cells	ART	100 μM	↓N/E-cadherin, MMP9, vimentin, EMT	[81]
	HepG2215 cells	DHA	100 μM	↓Migration ability;	[55]
	HCCLM3 and MHCC97H cells	ART	25–100 μM	↓N-cadherin, MMP2, MMP9, Snail, E-cadherin	[60]
	HCCLM6 cells	DHA	50–100 μM	↓FN1, ITGB1	[59]
	HepG2 and SMMC-7721 cells	ART	12.5–75 μM	↓MMP2; ↑TIMP2	[82]
	HepG2 cells	ART	6.25–50 mmol/L	↓VEGF, PIGF	[83]
	HepG2 and Huh7 cells	ART	25 μM , 125 μM	↓Cell migration	[84]
Effects on hepatic stellate cells					
Liver fibrosis	Mouse HSCs and LX-2 cells	ART	25–75 μM	↓Cell vaibility, GSH, GPX4, NADPH, α-SMA, collagen 1, fibronectin, desmin, FTH1, NCOA4, p62; ↑cell death, Fe^2+^ accumulation, LPO, MDA, ROS, ferroptosis, LC3; mitochondria morphology change	[87]
	HSC-T6 cells	ARM	10–40 ug/ml	↓α-SMA, collagen 1, fibronectin, TGF-β, PDGF-β, EGF, cell viability, GSH, NADPH, Gpx4, SLC7A11; ↑cell death, Fe^2+^, ROS1, ferroptosis, p53; mitochondria morphology change	[88]
	LX-2 cells	ARM	10–40 ug/ml	↓cell activation; ↑cell death, Fe^2+^, ROS, MDA, LPO, ferroptosis, IRP2	[89]
	LX-2 cells	ART	12.5–50 ug/ml	↓cell activation, α-SMA, collagen 1, p-Akt, p-FAK, p-GSK-3β	[64]
	LX-2 cells	ART	350umol/L	↓hydroxyproline; ↑ceramide synthase protein, ceramide, PPAR-γ, caspase-3	[90]
	LX-2 cells	ART	50–200 μM	↓cell viability, Col1a1, Col3a1, OGDH, CS, IDH2, mitochondrial function, NDUFB8, UQCRC2; ↑Cell apoptosis	[91]
Effects on cell autophagy					
Liver fibrosis	Primary rat HSCs	DHA	5–20 μM	↓P62, inflammatory factors, GSH/GSSG; ↑autophagosome, autophagic flux, LC3-II, ROS, p-JNK1/2	[54]
	Primary rat HSCs	DHA	5–20 μM	↑autophagosome, p-ULK1, p-mTOR, Atg6/Beclin1, LC3-II, p53, GATA6, cell senescence	[99]
HCC	HepG2215 cells	DHA	100 μM	↓p-AKT, p-mTOR, p-p70S6K, p-4EBP1; ↑LC3, p-AMPK, p62	[55]
	HepG2215 cells	DHA	21.5 μM	↓p62/SQSTM1; ↑LC3B-II	[56]
Effects on chemsensitization to chemotherapeutic agents					
HCC	SM-7721, SK-hep1 cells	ART	50 μM	Chemosensitize with Sor, ↓IC50, cell viability, p-RAF, p-ERK, p-AKT, p-mTOR; ↑cell apoptosis, p-PARP	[93]
	HepG2 cells Huh7 cells	ART	25 μM 125 μM	Chemosensitize with Sor, ↓IC50, VEGFR2; ↑cell apoptosis, cleaved caspase-9, cleaved PARP	[84]
	HepG2 and Hep3B cells	ART and DHA	10 μmol/L	Chemosensitize with gemcitabine, ↓cell survival	[51]
	Hep3B cells	DHA	5 μM	Chemosensitize with ADM, ↓cell viability, clone, P-gp, p-ERK1/2, p65; ↑cell apoptosis	[95]

### Effects of artemisinin and its derivatives in vivo

Next, we will explore the effects of artemisinins on hepatitis, AFLD, NAFLD, liver fibrosis, and liver cancer, as well as the underlying mechanisms, including oxidative stress, hepatic inflammation, immunoregulation, and tumorigenesis (Table 3).

**Table 3:** Pharmacological effects of artemisinin and its derivatives on hepatic diseases in vivo

Hepatic disease	Animals/stimulation (model)	Drug	Dosage/administration route	Variations	Ref
Effects on oxidative stress and inflammation					
Liver injury	Newborn piglets / IUGR	DHA	80 mg/kg; orally	↓MDA, H2O2, PC, 8-OhdG, GSSG, GSSG:GSH; ↑T-SOD, T-AOC, GSH, Nrf2, HO-1	[104]
Liver injury	Adult male Wistar rats/I/R insult	ART	50 mg/kg; i.p	↓NLRP3, NF-κB, TNF-α, IL-18, IL-1β, HMGB1, IL-6	[105]
Liver fibrosis	Sprague–Dawley rats/multiple pathogenic factors	ART	28.8 mg/kg; orally	↓TNF-α, IL-6, NF-κB p65	[108]
Autoimmune hepatitis	Male Balb/c mice/ConA	ART	27, 54, 108 mg/kg; oral gavage	↓IL-6, IL-1β, TNF-α, IFN-γ, IL-17, NF-κB p65, p-JNK, p-ERK, p-p38; ↑IL-10	[109]
Cholestatic hepatitis	Adult male mice/lithocholic acid	Artemisinin	100 mg/kg; oral gavage	↑GSH, MRP2, CRA, FXR	[110]
Liver fibrosis	Male Sprague–Dawley rats/BDL	DHA	3.5, 7, 14 mg /kg; i.p	↓liver/body weight, serum AST, ALT, TNF-α, IL-6	[111]
Liver injury	Newborn piglets/IUGR	DHA	80 mg/kg/d; orally	↓AST/ALT ratio, TNF-α, IL-6, IL-1β, IFN-γ	[113]
NAFLD	Male C57BL/6 J mice/High-fat diet	AA extract	400 mg/kg/day; orally	↓COX-2, TGF-β1, HMGB1	[114]
Liver fibrosis	Male Sprague–Dawley rats/CCl4	DHA	3.5, 7, 14 mg /kg; i.p	↓NF-κB, TNF-α, IL-6, IL-8, NLRP3, IL-1β; ↑IL-4, IL-10, ROS, JNK1/2	[54]
AFLD	Male Sprague–Dawley rats/alcohol	DHA	7 mg/kg/day; i.p	↓TNF-α, NF-κB, NLRP3	[120]
Effects on lipid metabolism					
NAFLD	Male C57BL/6J mice/High-fat diet	AA extract	400 mg/kg/day; orally	↓body weight, liver weight, TG, ChREBP, SREBP1; ↑p-ACC	[114]
NAFLD	Female SWISS mice/HFD + LPS	Artemisinin	0.25 mg/kg/day; i.p	↓NOS2; ↑adipolysis	[118]
Liver injury	Newborn piglets/IUGR	DHA	80 mg/kg/d; orally	↓TC, VLDL-C, NEFA, FAS, ACCβ, SREBP-1; ↑HDL-C, LPL, HL, TL, AMPKα, SIRT1, CPT-1	[113]
AFLD	Male Sprague–Dawley rats/alcohol	DHA	7 mg/kg/day; i.p	↓TC, TG, FAS, SREBP-1c; ↑PPAR-α, CPT1, FXR	[120]
AFLD	Male ICR mice/alcohol	DHA	20 mg/kg/day; i.p	↓SREBP-1c, SCD1; ↑PPAR-α, FGF21, FSP27, VNN1	[122]
AFLD	Male ICR mice/alcohol	DHA	7 mg/kg/day; i.p	↓lipin-1β, FAS, SREBP-1c, SCD; ↑PPAR-α, CPT1α	[123]
Effects on cell apoptosis					
Liver fibrosis	Male Sprague–Dawley rats/BDL	DHA	3.5, 7, 14 mg /kg; i.p	↓Bcl-2, p-ATK, p-PI3K; ↑HSCs apoptosis, Bax, cleaved caspase-9, cleaved caspase-3, cleaved PARP-1, Cyt c	[111]
AFLD	Male ICR mice/alcohol	DHA	20 mg/kg/day; i.p	↓hepatocytes apoptosis, caspase-9/-3, PUMA, Bim	[122]
AFLD	Male ICR mice/alcohol	DHA	7 mg/kg/day; i.p	↓hepatocyte lipoapoptosis, caspase-9/3; ↑Bcl-2	[123]
Liver injury	Adult male Wistar rats/I/R insult	ART	50 mg/kg; i.p	↓hepatocytes apoptosis, Bax; ↑Bcl-2	[105]
Liver cancer	Mice/Subcutaneous injection of Hep G2 cells	DHA	20 mg/kg; i.p	↓weights of Hep G2 xenografts, Mcl-1, ↑Bak, cleaved caspase 3	[50]
Liver cancer	Mice/Subcutaneous injection of Hep G2 cells	DHA	5 mg/kg; i.p	↓Mcl-1, p-ERK, ↑PARP, necrosis	[129]
Effects on hepatic fibrogenesis					
Liver fibrosis	Sprague–Dawley rats/multiple pathogenic factors	ART	28.8 mg/kg; orally	↓TLR4, MyD88, TGF-β1, collagen, α-SMA	[108]
Liver fibrosis	Male Sprague–Dawley rats/bile duct ligation	DHA	3.5, 7, 14 mg/kg; i.p	↓HSC activation, PDGF, PDGF-βR, TGF-βRI, TGF-βRII, EGFR, p-ERK, α-SMA, α1 (I) collagen, fibronectin, TIMP-1; ↑PPARγ	[52]
Liver fibrosis	Male Sprague–Dawley rats/BDL	DHA	3.5, 7, 14 mg /kg; i.p	↓Collagen, α-SMA, α1(I) procollagen and fibronectin, TGF-βRII, PDGF-βR, EGFR	[111]
Liver fibrosis	Male ICR mice/CCl4	ART	50, 100, 200 mg/kg/day; i.p	↓α-SMA, collagen1, fibronectin, desmin	[87]
Liver fibrosis	Male ICR mice/CCl4	ARM	5, 10, 20 mg/kg; i.p	↓hydroxyproline, α-SMA), α1(I) collagen, fibronectin, PDGF-βR, EGFR	[88]
Liver fibrosis	Male wistar rats/bovine serum albumin	ART	3.2, 9.6, 28.8, 53.1 mg/kg; oral gavage	↓collagen, α-SMA, type I collagen, MMP-2, MMP-9; ↑ MMP-13	[126]
Liver fibrosis	Male ICR mice/CCl4	DHA	7 mg/kg/day; i.p	↓α-SMA, fibrotic, nodular; ↑FXR	[127]
Effects on autophagy					
Liver fibrosis	Male Sprague–Dawley rats/CCl4	DHA	3.5, 7, 14 mg /kg; i.p	↓p62, ↑autophagosome, LC3-II, ROS,	[54]
Liver fibrosis	Male Sprague–Dawley rats/CCl4	DHA	3.5, 7, 14 mg /kg; i.p	↓p-mTOR; ↑HSC senescence, p53, p16, GATA6, autophagosome, LC3-II, p-ULK1	[99]
Effects on ferroptosis					
Liver fibrosis	Male ICR mice/CCl4	ART	50, 100, 200 mg/kg/day; i.p	↓GSH; ↑Fe2 + , lipid ROS, Ptgs2	[87]
Liver fibrosis	Male ICR mice/CCl4	ARM	5, 10, 20 mg/kg; i.p	Small and ruptured mitochondria; ↓Gpx4, SLC7A11; ↑ROS1, P53	[88]
Effects on metastasis					
Liver disease	Male athymic BALB/c nu/nu mice/orthotopic xenografts	Artemisinin	0, 50, 100 mg/kg; oral gavage	↓lung tumors	[82]

i.p. Intraperitoneal injection

### Effects of artemisinins on hepatic oxidative stress and inflammation

The anti-oxidative effect of DHA has been demonstrated in weaned piglets with intrauterine growth retardation (IUGR). Previous studies have shown that IUGR is associated with cellular oxidative damage [102, 103]. DHA (80 mg/kg) effectively increased glutathione (GSH) concentrations and the activity of T-superoxide dismutase (T-SOD), and decreased the concentrations of malondialdehyde (MDA), H_2_O_2_, protein carbonyl (PC), 8-hydroxy-2’-deoxyguanosine (8-OhdG), oxidized glutathione (GSSG), and GSSG:GSH ratio in the liver. Further, DHA attenuated oxidative stress due to IUGR by activating the Nrf2/ARE/HO-1 signaling pathway [104]. Oxidative stress is one of the core molecular mechanisms for hepatic ischemia/reperfusion (I/R) injury. ART (50 mg/kg, intraperitoneal injection) significantly attenuated hepatic I/R injury by increasing GSH and SOD, which combat oxidative stress, and reducing the level of MDA, a marker of lipid peroxidation, at the beginning of the reperfusion period [105].

Additionally, emerging evidence revealed that artemisinins regulate signaling pathways and the production of inflammatory cytokines and chemokines to reduce hepatic inflammation and injury. Toll-like receptor 4 (TLR4) plays a crucial role in regulating inflammation and is unique for containing lipopolysaccharide (LPS). By triggering myeloid differentiation primary response protein 88 (MyD88), NF-κB protein is activated, leading to the release of inflammatory factors [106, 107]. An in vivo study demonstrated that ART downregulated the expression of TLR4, MyD88, and NF-κB at both the mRNA and protein levels, and reduced proinflammatory factors TNF-α and IL-6 in a hepatic injury model induced using several pathogenic factors [108]. Research has shown that ART (27, 54, and 108 mg/kg) exhibits anti-inflammatory properties and plays a protective role against concanavalin A (Con A)-induced inflammatory activity and autoimmune injury in mouse livers by decreasing the serum levels of aspartate transaminase (AST) and alanine transaminase (ALT) and reducing production of pro-inflammatory cytokines, such as IL-6, IL-1β, TNF-α, IFN-γ, and IL-17. This effect was mediated mainly by inhibiting the activation of NF-κB and MAPK pathways [109]. A later study suggested that oral exposure to lithocholic acid (LCA) caused liver injury, but the anti-inflammatory activity of artemisinin extract in liver tissues increased the expression of GSH in a LCA-induced mouse model [110]. Chen and colleagues reported that DHA treatment improved hepatic necrosis and infiltration of inflammatory cells, thus playing a protective role in bile duct ligation (BDL)-induced injury in a rat model. Moreover, DHA reduced the levels of serum ALT, AST, TNF-α, and IL-6 [111]. IUGR reportedly induces inflammation [112]. Levels of serum AST and ALT and the AST/ALT ratio were high in IUGR-affected piglets, which indicated liver damage. Additionally, the levels of IFN-γ, TNF-α, IL-1β, and IL-6 were found upregulated in the livers of IUGR-affected piglets. However, DHA supplementation reduced the expression of proinflammatory cytokines and played an important role in protecting hepatic function [113]. Another study showed that Artemisia annua leaf (AA) extract improved hepatic inflammation by reducing the expression of high-mobility group box 1 (HMGB1) and cyclooxygenase-2 (COX-2) in high-fat diet (HFD)-fed mice [114].

**Fig. 1:**
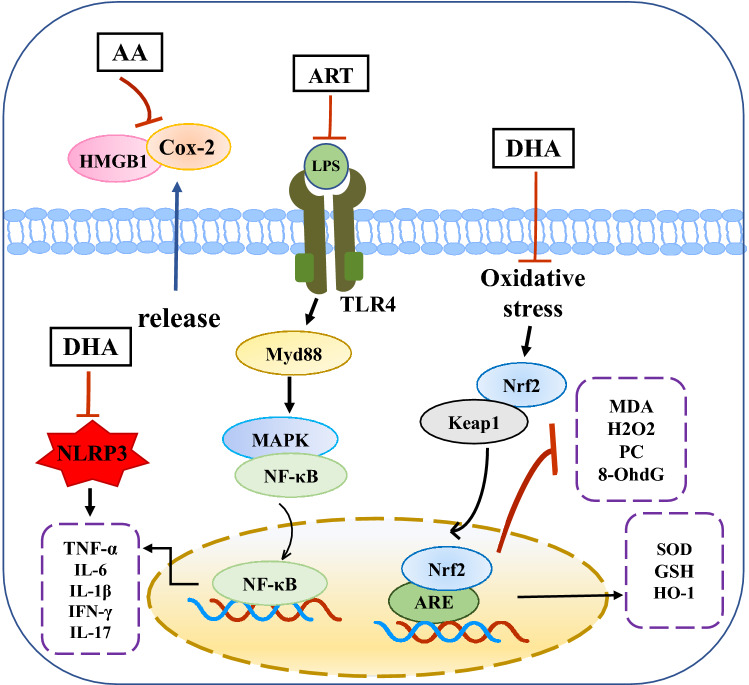
Effects of artemisinin and its derivatives on hepatic oxidative stress and inflammation

Several studies have confirmed the role of autophagy as a defense mechanism against inflammation-related diseases [115, 116]. Zhang and colleagues found that DHA not only inhibited the production and release of inflammatory cytokines (NF-κB, NLRP3, TNF-α, IL-1β, IL-6, and IL-8), but also promoted the expression of anti-inflammatory factors (IL-4 and IL-10) in a CCL_4_-induced rat model. Furthermore, DHA ameliorated inflammation by inducing autophagy in activated HSCs. Importantly, DHA induced autophagy in HSCs by promoting ROS generation and c-Jun N-terminal kinase (JNK)1/2 activation [54]. Proposed underlying mechanisms are shown in Fig. 1.

### Effects of artemisinins on hepatic lipid metabolism (fatty liver diseases)

**Fig. 2:**
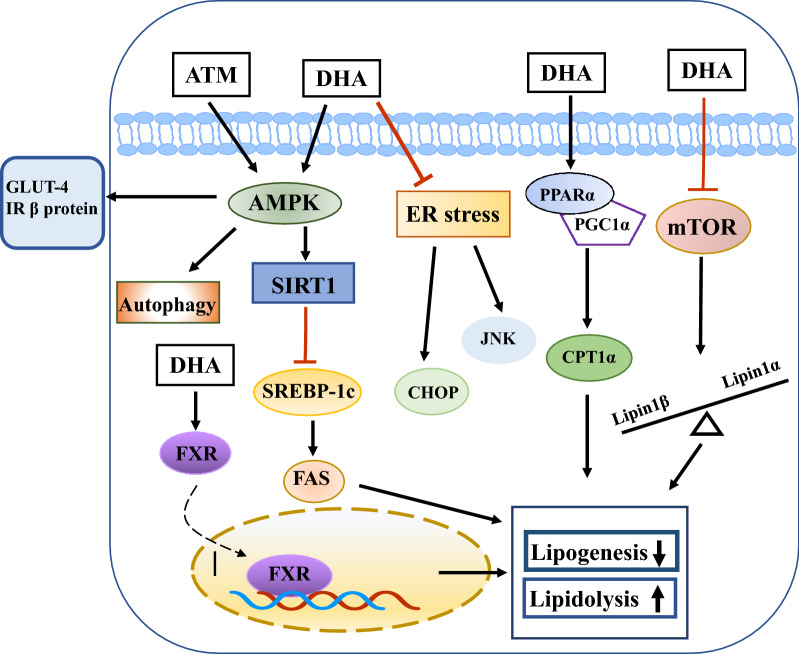
Effects of artemisinin and its derivatives on hepatic energy metabolism

In recent years, evidence has accumulated indicating that metabolic syndromes, such as obesity, insulin resistance, diabetes, and hyperlipidemia, and AFLD caused by excessive alcohol consumption are major causes of chronic liver diseases and even HCC [117]. Artemisinin and its derivatives have shown defensive effects on hepatic lipid metabolism disorders (Fig. 2).

AA extract administration significantly decreased the expression of p-acetyl-CoA carboxylase (ACC), carbohydrate-responsive element-binding protein (ChREBP), and sterol regulatory element-binding protein 1 (SREBP1), the regulators of lipid synthesis, and improved hepatic triglyceride (TG) levels, body weight, and insulin resistance in HFD-fed mice [114]. A study reported that 0.25 mg/kg artemisinin demonstrated beneficial effects in inflammatory obese mice by decreasing adipose stores and NAFLD/ non-insulin-dependent diabetes mellitus (NIDDM) in hepatic tissue [118]. In IUGR-affected piglets, the serum total cholesterol (TC), very-low-density lipoprotein cholesterol (VLDL-C), and non-esterified fatty acid (NEFA) concentrations were high, while total lipase (TL), hepatic lipase (HL), and lipoprotein lipase (LPL) activities of the liver were decreased. However, DHA treatment reversed the above phenomena by promoting the adenosine 5’-monophosphate-activated protein kinase (AMPK)/SIRT1 signaling pathway in the liver to ameliorate lipid metabolism [113]. Interestingly, ARM was found to reduce liver fat vacuoles, improve hepatic insulin resistance, and decrease fat deposition in db/db mice [119]. Chronic, abundant alcohol consumption is a significant cause of lipid metabolism disorders. Xu and colleagues confirmed that DHA eliminated the alcohol-induced expression of proinflammatory factors, such as TNF-α, NF-κB, and NLRP3. DHA administration also reduced intrahepatic CD45-, F4/80-, and CD68-positive cells and monocyte chemotactic protein-1 (MCP-1) expression. On the contrary, DHA treatment decreased serum levels of TG, TC, and low-density lipoprotein cholesterol (LDL-C), which further prevented fat deposition. The specific farnesoid X receptor (FXR) antagonist Z-guggulsterone reduced DHA-induced effects significantly [120]. Activation of the endoplasmic reticulum (ER) stress-mediated mitochondrial pathway also plays an important role in the pathogenesis of AFLD. ER stress induced by alcohol promoted the expression of key lipogenic enzyme SREBP-1c, leading to increased fat synthesis and resulting in lipid metabolism disorders [121]. Furthermore, Chen and colleagues demonstrated that DHA (5, 10, 20 µM) prevents ER stress and mitochondrial apoptotic pathway activation, and inhibits JNK activation and expression of C/EBP homologous protein (CHOP). DHA attenuated alcohol-induced lipid accumulation in mouse livers by suppressing stearoyl-CoA-desaturase 1 (SCD1), Fas, and SREBP-1c, while increasing the expression of PPAR-α and carnitine palmitoyltransferase 1A (CPT-1A), the regulator of fatty acid oxidation [122]. Additionally, a previous study reported that DHA regulated nucleocytoplasmic shuttling of lipin-1 to alleviate alcohol-induced liver impairment in mice [123].

### Effects of artemisinins on liver fibrosis

**Fig. 3:**
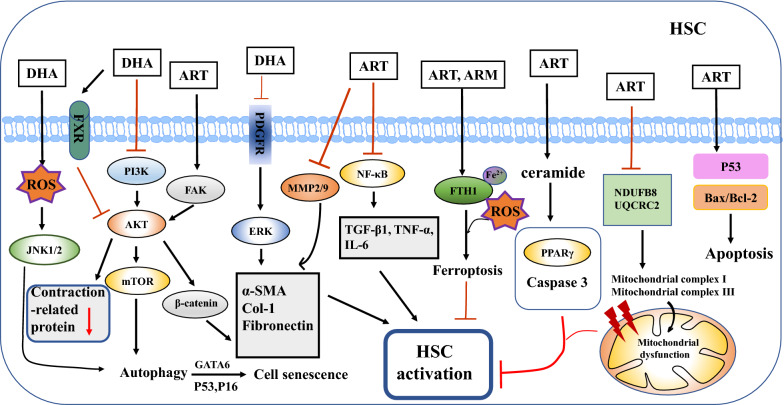
Effects of artemisinin and its derivatives on liver fibrosis

Liver fibrosis, a decompensated repair process following many chronic injuries, is characterized by the activation of HSCs, accumulation of ECM, and hepatic inflammation. Liver fibrosis often develops into cirrhosis, liver failure, portal hypertension, and HCC, and therefore urgently requires effective treatment [124, 125]. The effect of artemisinins on liver fibrosis and possible molecular mechanisms of action are summarized below (Fig. 3). ART (28.8 mg/kg) treatment decreased TNF-α, IL-6, α-SMA, TLR4, MyD88, NF-κB, and TGF-β1 levels in rats, demonstrating that it may ameliorate hepatic fibrosis by inhibiting liver inflammation [108]. In addition, previous studies showed that DHA disrupts HSC proliferation and promotes HSC apoptosis to prevent the progress of liver fibrosis in a BDL rat model [52, 111]. A recent study verified that ART induced ferritinophagy-mediated ferroptosis in HSCs in a CCL_4_-induced mouse model. Chloroquine inhibits ART-induced anti-fibrosis functions by eliminating ferritinophagy [87]. More importantly, p53, a tumor suppressor, was observed upstream of HSC ferroptosis, indicating that ARM alleviated hepatic fibrosis by mediating p53-dependent ferroptosis [88]. Another study found that DHA could treat liver fibrosis by inducing HSC autophagy [54]. Furthermore, DHA may promote HSC senescence by inducing the generation of GATA binding protein 6 (GATA6), in which the activation of autophagy plays an important role [99]. Meanwhile, ART prevented bovine serum albumin-induced liver fibrosis by decreasing hydroxyproline levels and inhibiting MMP-2, MMP-9, α-SMA, and type I collagen expression [126]. Interestingly, artemisinins not only ameliorated fibrosis by inhibiting HSC activation, but also treated portal hypertension via inhibition of HSC contraction. Xu and colleagues found that DHA inhibited HSC contraction by activating FXR and restraining contractile regulators, such as sphingosine-1-phosphate receptor 2 (S1PR2), phospho-myosin phosphatase target subunit 1 (p-MYPT1), rho-associated kinase (ROCK), myosin light chain kinase (MLCK), and phospho-myosin light chain (p-MLC) in rat HSCs [127].

### Effects of artemisinins on liver cancers

**Fig. 4:**
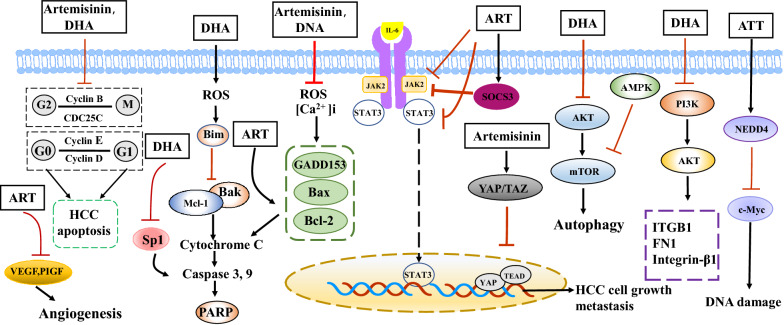
Molecular targets of artemisinin and its derivatives in liver cancer

HCC is the primary type of liver cancer, with high incidence and mortality rates [128]. However, treating HCC is still a clinical challenge with poor prognosis due to tumor metastasis, chemoresistance, and recurrence after surgery. Increasing evidence has shown that artemisinin and its derivatives have anti-tumor effects (Fig. 4). Zhang and colleagues found that 20 mg/kg DHA largely reduced the weights of HepG2 xenografts in mouse models, which was accompanied with the upregulation of Bak and caspase 3 and downregulation of Mcl-1 [50]. Additionally, DHA decreased cancer cells and induced apoptosis and inflammatory cell infiltration in tumor sections [129]. Another study reported that, compared with the control group, ART treatment reduced the number of tumors in the lungs of mice after liver inoculation with HepG2 tumor tissue, which indicated that ART possibly inhibited metastasis of HCC in vivo [82]. Recently, research has indicated that the IL-6/JAK/STAT signaling pathway was involved in the development and progression of HCC [130]. ART (25 mg/kg bodyweight) significantly prevented nitrosodiethylamine-induced hepatocarcinogenesis by downregulating the expression of IL-6, GP130, JAK-2, and STAT-3(pY705) in rats [131]. Interestingly, cellular-myelocytomatosis viral oncogene (c-Myc) reportedly protects human tumor cells from DNA damage by inducing the expression of topoisomerases. However, by promoting the expression of the c-Myc E3 ligase neural precursor cell expressed developmentally downregulated gene 4 (NEDD4), artemisitene (ATT) treatment destabilized c-Myc in tumor cells and damaged the DNA of tumor cells, thus playing an anti-tumor role [132].

### Metabolism of artemisinins and drug interactions

The liver is the primary organ of drug metabolism and is rich in various enzymes required for drug metabolism. Among them, the cytochrome P450 (CYP) enzyme family is the most important and plays a notable role in drug metabolism, cell metabolism, and homeostasis [133]. CYP enzymes involved in drug metabolism include, but are not limited to, CYP1A2, CYP2B6, CYP2C9, CYP2C19, CYP2D6, CYP2E1, and CYP3A4. Research has indicated that artemisinin was mainly metabolized into its active metabolite DHA in liver microsomes [134]. For instance, ARE was metabolized into DHA by CYP3A4, CYP2B6, and CYP3A5, and ART was metabolized to DHA by CYP2A6 [134–136]. Interestingly, the oral clearance rate of artemisinin on the last day of multiple administrations was significantly higher than that on the first administration, indicating that artemisinin can induce an auto-induction phenomenon, partly due to the induction of CYP2B6 [137]. Furthermore, a recent study demonstrated that auto-induction elimination caused by artemisinin might be related to intestinal first-pass effect, likely by inducing CYP3A4 and CYP2B6 in the intestine [138]. Therefore, the route of administration should be considered when artemisinin is used. Artemisinins play a significant role in the induction or inhibition of CYP enzymes, which is the main mechanism of drug interaction. We previously found that artemisinin, ARM, and ARE significantly decreased the metabolic elimination of carbamazepine from livers through the inhibition of hepatic CYP3A4 enzyme activity in rabbits [139]. Furthermore, there was also an interaction between artemisinins and other drugs, such as efavirenz, protease inhibitors, and S-mephenytoin [134, 137, 140]. The potential drug-drug interactions were mainly generated by regulation of CYP3A, CYP2B6, and CYP2C19. Apart from drugs, artemisinin also weakened the metabolism of caffeine in healthy subjects by inhibiting CYP1A2 activation [141]. The above findings suggest that drug-drug interactions must be considered when administering artemisinins.

### Toxicology

Thousands of patients have been treated with artemisinins to combat malaria, and no major adverse effects have been reported yet. Nevertheless, this alone is not strong enough evidence for the drug’s safety. A large meta-analysis including thousands of patients with malaria reported neutropenia, prolonged QT interval, and elevated liver enzymes in 1.3%, 1.1%, and 0.9% of all cases, respectively [142]. Deeken et al. identified that the maximum tolerated dose of intravenous ART was 18 mg/kg in patients with advanced solid tumors. Interestingly, patients receiving the minimum dose of intravenous ART (8 mg/kg) showed distinct infusion reactions during the follow-up treatment cycle [143]. Recently, some case reports also revealed that the use of artemisinins led to unwanted adverse events. A patient ingesting *A. annua* tea as a chemoprophylaxis against malaria suffered from severe acute cholestatic hepatitis, combined with increased liver enzyme and liver inflammatory activities [144, 145]. Further, we found that the association of ART with other drugs led to unexpected adverse events. In one of the cases reported, a glioblastoma multiforme (GBM) patient treated with temozolomide, ART, and Chinese herbal medicines (*Coptis chinensis*, *Siegesbeckia orientalis*, *Artemisia scoparia*, and *Dictamnus dasycarpus*) developed hepatotoxicity [146]. Though single drug use may induce hepatotoxicity, the combination therapy resulted in significantly increased liver enzyme activities. Another case of a GBM patient was reported; the patient received a combination of dichloroacetate and ART after failed temozolomide treatment and suffered from hepatic damage and bone marrow toxicity, and died a few days later [147]. The compassionate use of dichloroacetate/ART cannot be recommended in the clinical treatment of GBM. These extreme examples illustrate that, although artemisinins alone are considered to be well tolerated, their combination with other medications should be carefully administered. Of course, in many animal experiments, artemisinins exhibited neurotoxicity (cerebral parenchyma and nucleus damage), cardiotoxicity (bradycardia and QTc prolongation), hematotoxicity (leukopenia and thrombocytopenia), genotoxicity, embryotoxicity, and nephrotoxicity [148]. Most evidence reporting these adverse events was obtained from case reports, animal experiments, and reports of combination use of artemisinins with other medications, which introduces controversy when determining if artemisinins are toxic or not. However, it is undeniable that attention should be paid to the administration method, frequency, dosage, and combination of medications when using artemisinin and its derivatives in clinical practice.

## Conclusion and future perspectives

Compared with traditional therapies, the extraction of bioactive natural ingredients from Chinese herbal medicines has become increasingly popular, which may be due to the additional benefits in the prevention and treatment of chronic diseases, such as liver diseases. Artemisinin has played a huge role in the treatment of malaria in modern times. In recent years, artemisinins have also been widely used in the treatment of solid tumors [149, 150], respiratory diseases [32], and immune related diseases [151, 152]. However, the literature lacks systematic reports on the significance of artemisinin and its derivatives in the treatment of chronic hepatic disease. In this review, we summarized and provided an overview of the current knowledge of artemisinin and its derivatives as potential therapeutic targets for the treatment of liver diseases in vitro and in vivo. The important activities of artemisinins could be divided into four aspects: antioxidant, anti-inflammatory, pro-apoptotic, and carcinostatic. These four effects interact with each other to resist the onset and progression of cellular damage leading to the development of hepatitis, followed by cirrhosis and HCC. Moreover, it is important to note that artemisinins can play a role against HCC by antagonizing fibrogenesis at the end-stage of liver pathology and inhibit angiogenesis, invasion, and metastasis in the development of HCC. Additionally, artemisinin-induced autophagy, ferroptosis, and senescence of HSCs also constitute the mechanisms to treat liver diseases. Previous pharmacokinetic studies suggest that artemisinins are mainly metabolized by liver microsomes and can trigger auto-induction; thus, the half-life of artemisinin is short in vivo. Simultaneously, artemisinins affect the metabolism of other drugs by regulating enzymes related to metabolism. Some animal studies showed that certain doses of artemisinin produce toxic effects to varying degrees. Nevertheless, there are few toxic effects when artemisinins were used to treat malaria in humans. A possible reason for this is that smaller doses over longer periods are more toxic than larger doses over shorter periods. Therefore, this should be considered when artemisinin is used in the future.

Despite accumulating evidence for the application of artemisinins, their use as treatment for chronic hepatic disease in routine clinical practice is extremely limited. Due to the lack of randomized controlled clinical trials, the potential of artemisinins in the treatment of liver disease has not been well explored. The chemosensitization effect of artemisinins combined with chemotherapeutic agents requires further clarification. The evaluation of the therapeutic efficacy of artemisinins should be further standardized, especially in a NAFLD setting. To date, the treatment of chronic liver disease poses many difficult questions to be solved in clinical practice. Artemisinin, as a kind of safe and strong drug candidate, has provided a promising prospect for the treatment of chronic hepatic disease.

## Data Availability

Not applicable.

## Abbreviations

AFLD: Alcoholic fatty liver disease
NAFLD: Non-alcoholic fatty liver disease
HCC: Hepatocellular carcinoma
CCA: Cholangiocarcinoma
TCHM: Traditional chinese herbal medicine
ROS: Reactive oxygen species
ART: Artesunate
DHA: Dihydroartemisinin
ARM: Artemether
ARE: Arteether
NF-κB: Nuclear factor kappaB
AKT: Protein kinase B
TNF-α: Tumor necrosis factor-α
CDC25C: Cell division cycle 25 homolog C
E2F1: E2F transcription factor 1
CDK: Cyclin-dependent kinase
HSC: Hepatic stellate cell
PDGF: Platelet derived growth factor
NLRP3: NLR family pyrin domain containing 3
IL: Interleukin
IFN: Interferon
Bcl: B-cell lymphoma
Bax: Bcl-2-associated X
Apaf-1: Apoptotic protease activating factor-1
PARP: Poly ADP-ribose polymerase
MDM2: Mouse double minute 2
STAT: Signal transducers and activators of transcription
GADD153: Growth-arrest-and-DNA-damage-inducible gene 153
Bim: Bcl-2 interaction mediator of cell death
Mcl-1: Myeloid cell leukemia-1
Bak: Bcl-2 homologous antagonist/killer
MOMP: Mitochondrial outer membrane permeabilization
Puma: P53 upregulated modulator of apoptosis
EMT: Epithelial-to-mesenchymal transition
MMP: Matrix metalloproteinase
FN1: Fibronectin-1
ITGB1: β1-Integrin
PI3K: Phosphoinositide 3-kinase
TIMP: Tissue inhibitor of metalloproteinases
ECM: Extracellular matrix
Cdc42: Cell division cycle 42
VEGF: Vascular endothelial growth factor
PIGF: Placental growth factor
TGF-βR1: Tumor growth factor-β receptor 1
EGFR: Epidermal growth factor receptor
STUB1: STIP1 homology and U-box containing protein 1
IRP2: Iron regulatory protein 2
α-SMA: α-Smooth muscle actin
PPAR: Peroxisome proliferators-activated receptors
MAPK: Mitogen-activated protein kinase
mTOR: Mammalian target of rapamycin
ERK: Extracellular signal-regulated protein kinases
ADM: Doxorubicin
P-gp: P-glycoprotein
ULK1: Unc-like kinase 1
LC3: Light chain 3
p70S6K: P-ribosomal protein S6 kinase
4EBP1: 4E binding protein 1
LC3B: Light chain 3 beta
SQSTM1: Sequestosome 1
IUGR: Intrauterine growth retardation
GSH: Glutathione
T-SOD: T-superoxide dismutase
MDA: Malondialdehyde
PC: Protein carbonyl
8-OhdG: 8-Hydroxy-2’-deoxyguanosine
GSSG: Oxidized glutathione
I/R: Ischemia/Reperfusion
TLR4: Toll-like receptor 4
LPS: Lipopolysaccharide
MyD88: Myeloid differentiation primary response protein 88
Con A: Concanavalin A
AST: Aspartate transaminase
ALT: Alanine transaminase
LCA: Lithocholic acid
BDL: Bile duct ligation
AA: Artemisia annua leaf
HMGB1: High-mobility group box 1
COX-2: Cyclooxygenase-2
HFD: High-fat diet
JNK: C-Jun N-terminal kinase
ACC: Acetyl-CoA carboxylase
ChREBP: Carbohydrate-responsive element-binding protein
SREBP1: Sterol regulatory element-binding protein 1
TG: Triglyceride
NIDDM: NAFLD/ non-insulin-dependent diabetes mellitus
TC: Total cholesterol
VLDL-C: Very-low-density lipoprotein cholesterol
NEFA: Non-esterified fatty acid
TL: Total lipase
HL: Hepatic lipase
LPL: Lipoprotein lipase
AMPK: Adenosine 5’-monophosphate-activated protein kinase
MCP-1: Monocyte chemotactic protein-1
LDL-C: Low-density lipoprotein cholesterol
FXR: Farnesoid X receptor
ER: Endoplasmic reticulum
CHOP: C/EBP homologous protein
SCD1: Stearoyl-CoA-Desaturase 1
Fas: Factor related apoptosis
CPT-1a: Carnitine palmitoyltransferase 1a
GATA6: GATA binding protein 6
S1PR2: Sphingosine-1-phosphate receptor 2
p-MYPT1: Phospho-myosin phosphatase target subunit 1
ROCK: Rho-associated kinase
MLCK: Myosin light chain kinase
p-MLC: Phospho-myosin light chain
C-Myc: Cellular-myelocytomatosis viral oncogene
NEDD4: Neural precursor cell expressed developmentally downregulated gene 4
ATT: Artemisitene
CYP: Cytochrome P450
